# Public Concern about Haze and Ozone in the Era of Their Coordinated Control in China

**DOI:** 10.3390/ijerph20020911

**Published:** 2023-01-04

**Authors:** Yaling Lu, Yuan Wang, Yujie Liao, Jiantong Wang, Mei Shan, Hongqiang Jiang

**Affiliations:** 1 School of Environmental Science and Engineering, Tianjin University, Tianjin 300072, China; 2 The Center of Enterprise Green Governance, Chinese Academy for Environmental Planning, Beijing 100012, China; 3 Hebei Key Laboratory of Power Plant Flue Gas Multi-Pollutants Control, Department of Environmental Science and Engineering, North China Electric Power University, Baoding 071003, China

**Keywords:** public concern, haze, ozone, zoning, China

## Abstract

In China, due to the implementation of the Action Plan for Prevention and Control of Air Pollution (APPCAP), the concentrations of PM_2.5_ (fine particulate matter) and severe haze in most cities have decreased significantly. However, at present, haze pollution in China has not been completely mitigated, and the problem of O_3_ (ozone) has become prominent. Therefore, the prevention and control of haze and O_3_ pollution have become important and noticeable issues in the field of atmospheric management. We used the Baidu search indices of “haze” and “ozone” to reflect public concerns about air quality and uncover different correlations between level of concern and level of pollution, and then we identified regions in China that require public attention. The results showed that (1) over the last decade, the search index of haze had a rapid trend of variation in line with changes in haze pollution, but that of O_3_ had a relatively slowly increasing trend; (2) the lag days between the peaks of public concern and the peaks of air pollution became increasingly shorter according to daily data analysis; and (3) 96 polluted cities did not receive sufficient public attention. Although periods of heavily haze-polluted weather, which affects visibility, have generated much public concern, periods of slight pollution have not received enough public attention. Public health protection and environmental participation regarding these periods of slight pollution in China deserve appropriate levels of attention.

## 1. Introduction

The rapid development in China of urbanization and industrialization has led to severe air pollution, which typically is characterized by a heavy haze [1]. Haze is mainly derived from excess fine particulate matter (PM_2.5_) emitted from anthropogenic sources [2]. In 2013, in response to increasing air pollution, the Chinese government launched the Action Plan for Prevention and Control of Air Pollution (APPCAP). A target for lower PM_2.5_ concentrations in China by 2018 was included, according to a recommendation from the Ministry of Ecology and Environment [3]. However, this target does not meet WHO requirements [4], and mild haze still occurs frequently in China. Meanwhile, ozone (O_3_) pollution has become a new environmental issue in China. The O_3_ concentrations of 377 cities in mainland China were 138 µg/m^3^ in 2020, an increase of 12.6% since 2015. This issue has attracted the attention of the Chinese government. The focus of air pollution control in China has shifted, and coordinated control of PM_2.5_ and O_3_ is urgently needed following the successful implementation of APPCAP [5].

The control of O_3_ is more difficult than that of PM_2.5_ because its formation process is more complex, and it is less likely to be understood by the public compared with PM_2.5_, which is usually associated with conducive meteorological factors which lead to severe haze affecting atmospheric visibility [6,7]. Effective air quality management must be based on a solid public understanding of air pollution problems and solutions [8]. Although the public sometimes lacks information on environmental pollution and disasters, their basic concept of risk is heightened compared to that of experts [9]. Thus, an effective understanding of public awareness can affect the government’s decision making and facilitate the promulgation and implementation of a series of measures and policies.

Max Weber, a famous German sociologist, put forward the theory of the relationship between perception and action. He believed that social action could be described in this way; it is related to the behavior of others, and the process of action is oriented according to the actors or the intention of the actors [10]. This study aimed to carry out quantitative research into public behavior and public perceptions of atmospheric environmental management. Compared with traditional questionnaire surveys, the application of internet-based big data approaches allows some defects to be overcome, such as the limitation of the amount of information used and the quality of answers. This is conducive to the study of social perception and action theory from a more scientific and quantitative perspective. Public concern is an important factor affecting the public perception of environmental policies. As a kind of public good, the environment needs social attention to reflect its value and attract the attention of the government. Only by proposing stricter environmental protection policies can we encourage more research and development of air pollution prevention technology and protect the health of the public.

Public concern is an important factor affecting the public perception of environmental policies. From the 1960s to the 1970s, recognition grew that assessing the general public for their level of understanding regarding air pollution was necessary if air pollution was eventually to be eliminated [11,12,13]. Some studies have also found obvious associations between specific air pollutants and public concern about air quality [14,15,16]. In addition, some studies have been devoted to the public’s willingness to pay for pollution mitigation [17,18]. The methods for these studies have been mainly based on questionnaire surveys. The advent of big data has provided novel research methods for studies based on public perspectives. For instance, some studies have used big data to investigate the correlations between air pollutants and public concern [18,19]. In addition, some scholars have focused on the relationship between air quality and public concern about environmental protection using social-media-based perspectives [20,21,22]. At present, China is one of the largest countries affected by air pollution, and an effective understanding of air quality from the perspective of public concern will help to enhance environmental management.

Search engine technology is an important component of big data approaches, and search indices based on search engine data can reflect urban public concerns and perception of air pollution issues to a certain degree. In China, Baidu is the most popular Web search engine; it dominates over 80% of China’s search market [23], and has been applied in different research fields [18,24,25,26,27]. In a previous study, we also used a search index to reveal the important relationships between air quality monitoring data and public concern based on a case study of haze in China during the implementation of APPCAP [19]. However, with the coordinated control of PM_2.5_ and O_3_ in the era following APPCAP, we need to consider public concern about PM_2.5_ and O_3_ from a collaborative governance perspective.

As the search results for “haze” and “ozone” from the Baidu index platform are not commercialized, they can be considered to have higher credibility for indicating the public’s concerns about air quality. We use the Baidu search index of “ozone” and “haze” to analyze the variation in public concern about ozone and haze in the last decade, and to uncover different correlations in different stages between air quality and public concern. Then, we identified regions requiring public attention and present suggestions to improve public attention. Research on public concern about air pollutants will provide a reference for scientists studying air quality and enhance the government’s ability to respond proactively to environmental crises.

## 2. Materials and Methods

### 2.1. Data

The Baidu search index is the weighted sum of search frequency for a keyword in Baidu Web, which is based on the search volume of netizens on Baidu and takes keywords as the statistical object. The data began in January 2010 with three time scales: hourly value (24 h), daily mean value, and the average value of a user-defined time scale (more than one day); and three spatial scales: city, province, and country. We chose “haze” and “ozone” in Chinese as the search keywords because they are popular words used by the Chinese public to describe air quality. The search index values of “haze” were calculated via the following steps: typing the word “haze” in the Baidu index engine, then selecting the region names and setting the time parameters. Other keywords were investigated similarly.

Most haze is caused by excess fine particulates, i.e., PM_2.5_, emitted by anthropogenic sources [17]. Studies have proven that the PM_2.5_ concentrations are clearly higher in haze-conducive weather, and haze pollution can be reduced consistently with reductions in PM_2.5_ emissions [28,29]. Therefore, haze is characterized by the PM_2.5_ concentration. PM_2.5_ and ozone concentrations were taken from urban air quality monitoring data released by the China National Environmental Monitoring Centre, which have been available since 2013. In this paper, we used the daily mean value and monthly mean value of the PM_2.5_ concentration and the daily maximum 8 h average ozone concentration data. In addition, the population data were taken from the statistical yearbook of each province in mainland China.

### 2.2. Methods

#### 2.2.1. Public Concern Index for PM_2.5_ and Ozone

In this study, the public concern index (PI) is defined to represent the proportion of people concerned in one region, taking the air quality of this region as its weight. The PI of haze was calculated using Equation (1):

(1) $${\mathrm{PI}}_{\mathrm{haze}}={f(I}_{\mathrm{haze}}/P)\times (C0_{\mathrm{PM}2.5}/C_{\mathrm{PM}2.5})$$

where $I_{\mathrm{haze}}$ refers to the haze search index of a region in 2020, obtained from the Baidu index platform, and P refers to the population in the region. f() is the normalized function. $C0_{\mathrm{PM}2.5}$ refers to the annual level of PM_2.5_ concentration allowed by the national air quality standard, which is 35 µg/m^3^. $C_{\mathrm{PM}2.5}$ refers to the annual average concentration of PM_2.5_ in this region in 2020. The larger the ${\mathrm{PI}}_{\mathrm{haze}}$ is, the higher the public’s concern about haze and the higher their participation in the prevention and control of haze pollution and atmospheric environmental management.

Similarly, the PI of ozone was calculated using Equation (2):

(2) $${\mathrm{PI}}_{\mathrm{ozone}}={f(I}_{\mathrm{ozone}}/P)\times ({\mathrm{CO}}_{o3}/C_{o3})$$

where $I_{\mathrm{ozone}}$ refers to the ozone search index in 2020. ${\mathrm{CO}}_{o3}$ refers to the annual level of ozone allowed by the national air quality standard, which is 100 µg/m^3^. $C_{o3}$ refers to the annual average concentration of ozone in the region in 2020. Similarly, the larger the ${\mathrm{PI}}_{\mathrm{ozone}}$ is, the higher the public’s concern about ozone.

PI, a comprehensive public concern index for PM_2.5_ and ozone, was defined to represent the proportion of people concerned about haze and ozone, and the influence of air quality was considered.

(3) $$\mathrm{PI}=({\mathrm{PI}}_{\mathrm{haze}}+{\mathrm{PI}}_{\mathrm{ozone}})/2$$

The larger the PI, the higher the public’s concern about haze and ozone and the higher their participation in environmental protection and atmospheric environment management.

#### 2.2.2. Statistical Analysis

Correlation analysis is one of the main research methods used in this study. It is a statistical method used to research linear relationships between two variables and has been widely used in air pollution research [30,31]. The Pearson correlation coefficient has become the most used correlation coefficient to characterize the correlations of fixed-distance variables [32]. In this study, the Pearson correlation coefficient was applied to explore the relationships between air quality (PM_2.5_ and ozone concentration) and public concern about haze and ozone (search indices of haze and ozone). Its formula can be found in a previous study [19].

Lag correlation analysis is used to study the lag time and the correlations between the peaks of time series [33,34,35,36]. A time lag between haze or ozone pollution outbreaks and public concerns about haze or ozone may exist. Therefore, we examined whether there was such a lag phenomenon in the public response to serious haze or ozone pollution. According to the peak/end rule [37,38], we selected the typical peak periods in 2011–2020 for the daily lag correlation analysis. In addition, a cross-correlation function was applied to assess the lag time between PM_2.5_ or ozone concentration and the search index of haze or ozone, and the peak of cross-correlation was employed to show the lag time between the two data time series. More details can be found in a previous study [19].

## 3. Results

### 3.1. Temporal Analysis of Haze and Ozone and Public Concern about Them in China

**Figure 1:**
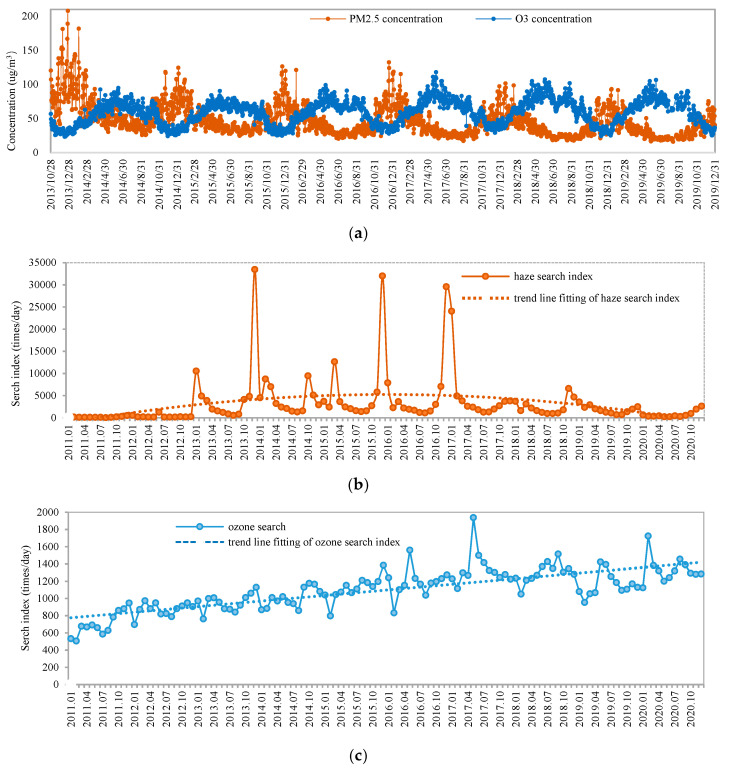
Concentrations of PM_2.5_ and O_3_ and the public concern about them: (**a**) concentrations of PM_2.5_ and O_3_; (**b**) search index of haze; (**c**) search index of ozone.

The daily concentrations of PM_2.5_ and ozone fluctuate seasonally in opposite directions (Figure 1a). In autumn and winter, haze occurs frequently because the PM_2.5_ concentration often exceeds the standard value; however, the ozone concentration is often relatively lower in these two seasons. In contrast, ozone pollution occurs frequently in summer, but the concentration of PM_2.5_ is lower. According to the average daily search indices of “haze” and “ozone” (times/day) from the Baidu search engine (Figure 1b,c) over the studied years, the public in China has paid increasing attention to these two environmental issues since 2011. Specifically, the haze search index increased rapidly in the first several years. Before January 2013, the haze search index was not high. It averaged 1262 searches per day in June 2012, which was the highest value in 2011 and 2012. In January 2013, the search index suddenly increased to 10,527 searches per day. This is because of the widespread haze in north China, which led to more than 7000 premature deaths and attracted extensive attention at home and abroad [39,40]. In December 2013, the index reached 33,432 searches per day, the highest value ever recorded. Moreover, there were two typical search index peaks associated with haze periods in 2015–2017, December in 2015 and December in 2016 [41,42]. Since then, with the effective resolution of the haze problem in most regions of China, the haze search index has decreased significantly. To some extent, this index shows that haze, as visible atmospheric pollution, is more concerning to the public; with the emergence of haze pollution, public attention increased; as the haze problem was gradually solved, the public paid less attention to it.

In contrast to the rapid variation trend of the haze search index, the ozone search index increased slowly with seasonal fluctuation. Generally, there were search peaks in autumn and winter from October to December and again in spring and summer from April to May. There were search troughs in the summer from July to August and early spring in February. However, the peak ozone search has appeared from May to September since 2016. China’s atmospheric pollution is dominated by particulate matter in winter and ozone in summer. In the process of coordinated collaborative governance of ozone and PM_2.5_, the focus is on ozone in summer and PM_2.5_ in winter.

Therefore, four typical peak periods of haze pollution, representing the four times at which the average concentrations of PM_2.5_ in cities of mainland China were the highest in the last 10 years, were selected for daily analysis (see Table 1). The results showed that the search index of haze increased progressively with increased PM_2.5_ concentration with a time lag of one day. In the first peak period (December 2013–January 2014), the maximum cross-correlation coefficient (CC) between the daily PM_2.5_ concentration and the haze search index was not significant, and the lag time was 3 days; however, the CC of the later peaks increased, and the lag time became shorter. By the fourth peak (December 2016), the CC reached a high correlation (CCmax = 0.893; Std. Error = 0.000), and the lag time was 1 day. Moreover, in the last two peak periods, the lag correlation coefficients for several consecutive days were significant, indicating that the public paid attention to haze for a longer time. The lag correlation analysis revealed that the public is likely to become increasingly sensitive to haze events as public environmental awareness improves.

Similarly to haze pollution, four typical peak periods of ozone pollution, which were the four times when the average concentrations of ozone in cities of mainland China were the highest in the last 10 years, were selected for daily analysis (see Table 2). Public attention to ozone is becoming increasingly immediate. Compared with the ozone pollution peak, the lag time of the public search peak of ozone in the first peak period (May 2016~June 2016) was 6 days; while in the last peak period (May 2019~June 2019), the lag time was 1 day. That means, compared with the ozone pollution peak, the lag time of the public search peak of ozone was shortened from 6 days from the first peak period to 1 day from the last one (see the red part in Table 2). When compared with Table 1, Table 2 shows that it is more difficult for the public to participate in the formulation of ozone pollution control policies and health protection strategies due to the invisibility of ozone. Therefore, decision makers need to strengthen publicity around ozone prediction, ozone pollution health protection, and public participation.

### 3.2. Spatial Analysis of Public Concern about Haze and Ozone and Zoning

**Figure 2:**
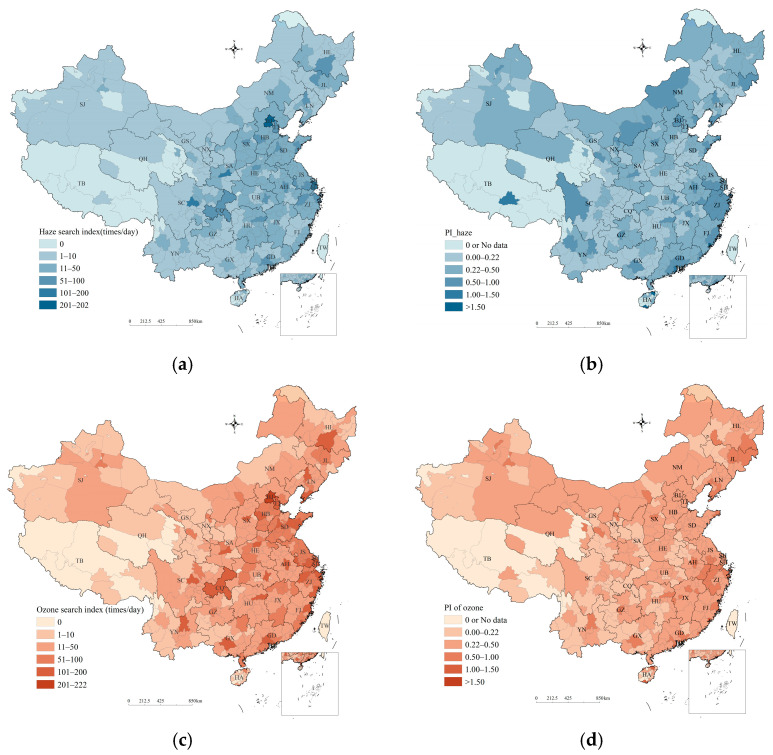
Search index and PI of haze and ozone in China: (**a**) haze search index; (**b**) PI_haze_; (**c**) ozone search index; (**d**) PI_ozone_.

The average daily search indices for haze and ozone in Chinese cities and the PI values of haze and ozone in 2020 are illustrated in Figure 2, which shows that the eastern and southern cities with more population had higher search indices (Figure 2a,c). Beijing, Shanghai, Xi’an, and Chengdu had higher haze search indices of more than 100 times/day. Beijing, Shanghai, Chengdu, Guangzhou, Shenzhen, Hangzhou, and Wuhan had ozone search indices higher than 150 times/day. The search indices for the two pollutants in less populated and economically underdeveloped areas in western China were smaller. Some cities in Tibet and Qinghai had a search index of 0. The Beijing–Tianjin–Hebei region and its surrounding areas in north China are the most frequently haze-polluted regions. Its surrounding areas, i.e., the junction region of Jiangsu–Anhui–Shandong–Henan in east China and the Fen-Wei Plains region and cities in Xinjiang in northwest China, are also haze-polluted regions. The average annual PM_2.5_ concentration in 49 cities, including Shijiazhuang, was above 50 µg/m^3^ in 2020. In addition, the Beijing–Tianjin–Hebei region and its surrounding areas in north China and the Yangtze River Delta region in east China are the most frequently ozone-polluted regions. The average annual ozone concentration in 64 cities, including Zibo, Tianjin, and Anyang, was above 160 µg/m^3^ in 2020. Although some cities suffered from serious haze and ozone pollution, the PI_haze_ according to Formula (1) and PIozone according to Formula (2) were not high (Figure 2b,d), indicating that the public paid little attention to the haze and ozone problem and may not participate enough in health and environmental protection. This situation was most obvious in Hebei and Shandong.

**Figure 3:**
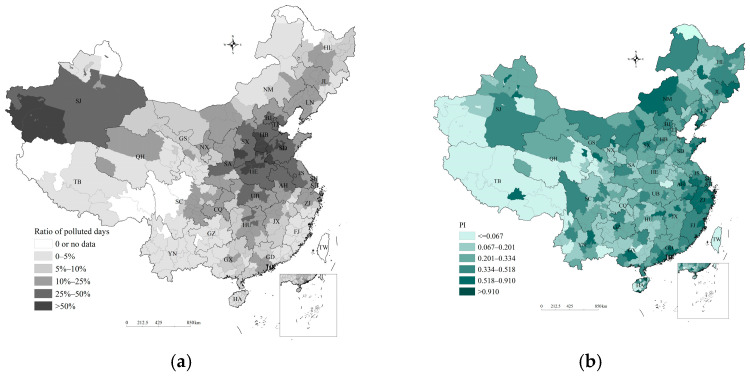
Air quality and PI in China: (**a**) proportion of polluted days; (**b**) PI.

Figure 3a shows the proportion of polluted days in every city in 2020. Beijing–Tianjin–Hebei and its surrounding areas in north China were the regions that most frequently exceeded the standard. Its surrounding areas, i.e., the Junction region of Jiangsu–Anhui–Shandong–Henan in east China and the Fen-Wei Plains region in northwest China, also often experience polluted weather. The air quality in Anyang, Jiaozuo, Handan, Shijiazhuang, Linfen, Xingtai, Liaocheng, Luoyang, Zhengzhou, Kaifeng, Zibo, Heze, and Jinan exceeded the national standard on more than 50 percent of all days in 2020. Monitoring data showed that PM_2.5_ and ozone were the main pollutants exceeding the standard in these regions. In addition, the air quality in some cities of Xinjiang was seriously below the standard due to sandstorms, which frequently increase PM_10_. The air quality of cities in southwest China was generally good, and there was seldom weather conducive to pollution. In theory, regions with a higher proportion of days exceeding the national standard should have a higher pollution search index. However, their distribution patterns were not completely consistent. This lack of consistency was most obvious in southern Hebei, western Shandong, and some cities in Xinjiang, which had lower PI but a higher ratio of polluted days (Figure 3b).

**Figure 4:**
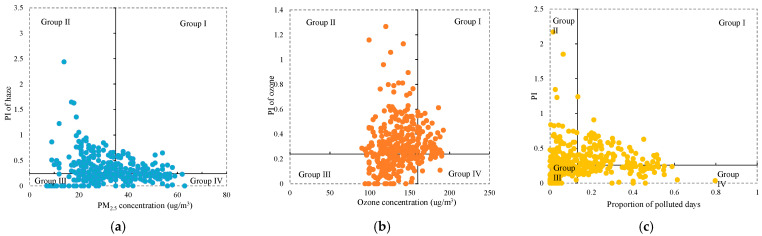
Four-quadrant classification of PI and air quality: (**a**) the four-quadrant classification of PI of haze and PM_2.5_ concentration; (**b**) the four-quadrant classification of PI of ozone and its concentration; (**c**) the four-quadrant classification of the comprehensive PI and proportion of polluted days.

To identify regions with more serious pollution but less public concern, all the cities were classified according to the following principles, as shown in Table 3. The classification results are shown in Figure 4. The cities in Group IV were the target cities. For PI_haze_ and haze pollution, there were 78 cities in Group IV, i.e., Tangshan, Handan, Xingtai, Cangzhou, and so on. For PI_ozone_ and ozone pollution, there were 21 cities in Group IV, i.e., Tianjin, Handan, Cangzhou, Hengshui, and so on. For the comprehensive PI and proportion of polluted days, there were 78 cities in Group IV, i.e., Handan, Xingtai, Cangzhou, Hengshui, and so on.

**Figure 5:**
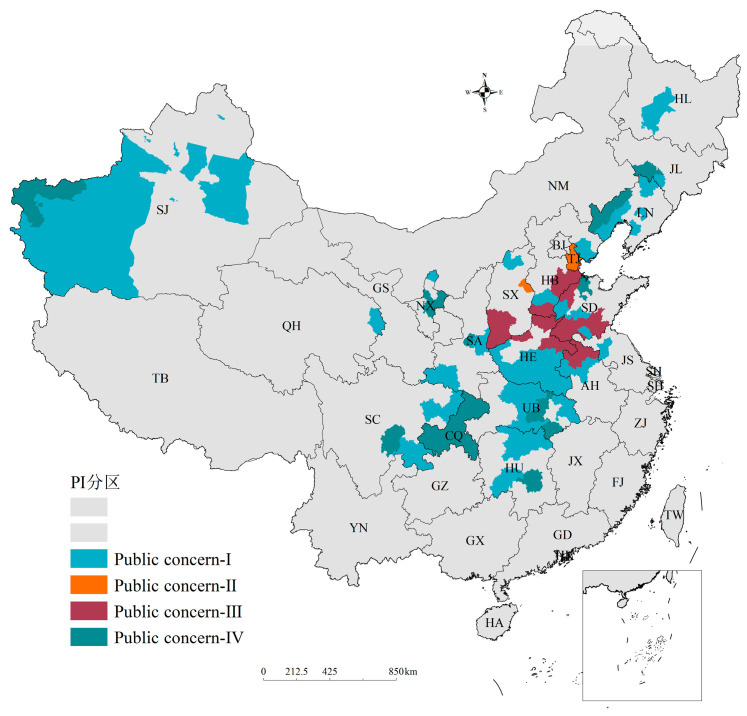
Public concern zoning against the background of PM_2.5_ and ozone joint prevention and control.

All of the cities in group IV were integrated for public concern zoning against the background of PM_2.5_ and ozone joint prevention and control. All the cities in this group have high pollution but little attention and should be identified as target cities for further research; meanwhile, the cities in the other groups have good air quality or enough public concern, or both. The colored areas in Figure 5 indicate regions in which the public needs to pay more attention to haze or ozone. We have labeled them the “public concern region” for short. There were 96 cities defined as “public concern regions”, which were divided into four categories. Tangshan, Xingtai, Suzhou, Jinzhou, and 55 other cities were zoned to Region Ⅰ. Members of the public in this region need to pay more attention to haze pollution. Tianjin and Yangquan were zoned to Region Ⅱ, where the public needs to pay more attention to ozone pollution. Nineteen cities belonged to Region III, where the public needs to pay more attention to both haze and ozone. Sixteen cities belonged to Region IV, where the region is potentially polluted but has insufficient public attention. According to this zoning, the public can be targeted to promote environmental health knowledge and encourage participation in the formulation of environmental protection policies.

## 4. Conclusions

Since 2011, due to frequent occurrences of haze, the US embassy, some communities, and NGOs have purchased their own PM_2.5_ monitors and have begun releasing unofficial data to citizens [43]. This has drawn growing public concern about haze in China. The Chinese public has begun to understand urban air quality and pollutants, and this under-standing promotes public participation processes. With the implementation of the air pollution prevention and control action plan and other measures, the focus of air pollution prevention and control in China has changed, and the public’s attention to air pollution also has new characteristics. Internet big data provides novel data sources for research on public views for environmental management. The main conclusions based on the big data used in this study are as follows:

(1) The search index of haze was relatively low before 2013, but after the large-scale haze event in China in January 2013, public concern about haze showed explosive growth. This is due to contributions from both severe air pollution events and growing public awareness. However, since 2017, due to the significant reduction in haze, the public’s concern about it has decreased significantly. The maximum value of the haze search index was 203,020 on 20 December 2016.

(2) Unlike the significant increase and rapid decrease in public concern about haze, the search index of ozone showed a slow rise. The maximum value of the ozone search index was 10,534 on 19 May 2017. In the past two years, searches for ozone have sometimes exceeded those of haze, although they have not increased explosively. Even at its highest, the maximum value of ozone searches was only 5% of that of haze searches. The invisibility of ozone makes it difficult for the public to participate in the formulation of ozone pollution control policies and health protection.

(3) Four typical peak periods of haze pollution and ozone pollution, representing the four times when the average concentrations of PM_2.5_ or ozone in mainland China were the highest in the last 10 years, were selected for daily analysis. According to daily data analysis, the lag times of public concern about haze and ozone have become shorter. Along with the improvement of public environmental awareness and environmental information disclosure, the public in China has become more sensitive to serious haze with a one-day lag.

(4) Multiple “public concern regions” were identified. Data from the Baidu platform showed that the eastern and southern cities with higher populations had higher search indices. The search indices in less populated and economically underdeveloped areas in western China were lower. The PI index is defined to represent the proportion of people concerned about haze or ozone in a region. It was obvious that in southern Hebei, western Shandong, and some cities in Xinjiang, PI was low, but the ratio of polluted days was high. To identify regions with more serious pollution but less public concern, we classified all the cities in China according to the same classification principles. The results showed that there were 96 cities classified as “public concern regions”, which can be targeted to promote environmental health knowledge, and in which the public can be encouraged to acquire air pollution protection knowledge and participate in the formulation of environmental protection policies.

Based on the above conclusions, suggestions for improving public participation in atmospheric environmental management are put forward:

Air quality is an important factor affecting public attention and search behavior related to relevant keywords on the search platform. The higher the concentration of air pollutants, the greater the number of searches. While air pollution is the basis of public concern, the media and others can also play a role. Active publicity by the media is required to improve the public’s participation in air pollution prevention and health protection. Another study [44] also suggests engaging citizens in the decision-making process to improve their political trust and publicizing knowledge of haze pollution to help the public acquire objective and scientific knowledge.

Moreover, due to the convenience of big data in atmospheric environment management, it is important to improve the application of big data in accurate atmospheric environmental management decision making. Therefore, in the process of environmental management, modern information tools should be used to obtain public suggestions. This will help the government to develop environmental indicators and policy measures that are more easily understood by the public. As a result, misalignment between public perception and the government on environmental governance can be largely mitigated.

## 5. Discussion

Damage to human health from haze has been proven by research [16]. Some researchers also suggest that short-term ozone exposure might increase the risk of ischaemic events, especially in individuals with severe vascular risk factors. They propose that an inflammatory reaction induced by ozone might be responsible for cardiac and cerebral ischaemic events [45,46]. How to raise the public’s awareness of invisible ozone pollution and maintain sufficient public attention to low haze pollution is an issue that needs further research. In addition to serious air pollution, other factors can also lead to an increase in public concern. Long-term public concern may be affected by other social factors (e.g., media campaigns, the improvement of environmental awareness, and the expansion of people’s use of search engines). On the one hand, the government can make efforts to publicize the harms of air pollution; meanwhile a more effective emergency plan can be developed to better understand the lag phenomenon of public concern about air quality to dispatch emergency teams more efficiently.

This study has the following points of significance. Firstly, analyzing the changes in public concern about haze and ozone and identifying the regions that need public attention are conducive to formulating corresponding measures to improve public concern and participation in atmospheric environmental policies. Secondly, the reasonable utilization of big data and search engines has several benefits for the study of public concern about air quality. For instance, the application of big data can measure the public concern about air pollution quantitatively, which can help the government to develop a better understanding of the environmental problems concerning the public (i.e., top-down approach). Therefore, the government can effectively formulate environmental policy and indicator measures that are more easily accepted and understood by the public. These actions will help the public to proactively participate in the related policy-making decisions about environmental problems (i.e., bottom-up approach). In addition, compared with traditional monitoring systems, search engines have certain advantages due to their convenience and high level of efficiency. With the rapid development of scientific technologies, more statistical approaches can be used to collect valuable information from big data.

There are some limitations and uncertainties in this study that need to be addressed. First, the search engine data have certain weaknesses associated with various factors, such as commercial interference and politics. Fortunately, “haze” and “ozone” are topics that can be freely discussed in China and receive little interference from the government, and they are also neutral words involving minimal commercial interest. Second, according to the State Statistical Bureau, there were 989 million internet users in China in 2020, accounting for 70.6% of the national population. In the future, research could attempt to combine big data with traditional questionnaire surveys to include people who do not use the internet. In China, Baidu is the largest search engine, accounting for more than 80% of China’s internet search market. This meant that this study excluded less than 20% of internet search behavior. Future research should consider those people who use other search engines. This can further advance the rigor of research methods and accuracy of results.

## Figures and Tables

**Table 1:** Lag correlation coefficient between public concern and PM_2.5_ concentration for different haze pollution peaks.

December 2013~January 2014 (Peak Time: 25 December)			December 2014~January 2015 (Peak Time: 4 January)			December 2015~January 2016 (Peak Time: 23 December)			December 2016 (Peak Time: 19 December)		
Lag	CC	Std. Error	Lag	CC	Std. Error	Lag	CC	Std. Error	Lag	CC	Std. Error
−7	0.181	0.433	−7	−0.064	0.781	−7	−0.198	0.390	−7	−0.487 *	0.025
−6	0.126	0.586	−6	0.212	0.355	−6	−0.214	0.351	−6	−0.577 **	0.006
−5	0.020	0.930	−5	−0.194	0.400	−5	−0.257	0.261	−5	−0.472 *	0.031
−4	−0.062	0.790	−4	−0.146	0.528	−4	−0.285	0.211	−4	−0.295	0.195
−3	−0.087	0.708	−3	−0.517 *	0.016	−3	−0.208	0.366	−3	−0.176	0.446
−2	−0.139	0.549	−2	−0.580 **	0.006	−2	−0.009	0.968	−2	−0.035	0.882
−1	−0.137	0.553	−1	−0.158	0.493	−1	0.249	0.277	−1	0.293	0.197
0	−0.008	0.972	0	0.405	0.069	0	0.541 *	0.011	0	0.719 **	0.000
1	0.086	0.711	1	0.376	0.093	1	0.671 **	0.001	1	0.893 **	0.000
2	0.100	0.666	2	−0.016	0.944	2	0.661 **	0.001	2	0.710 **	0.000
3	0.125	0.590	3	−0.387	0.083	3	0.437 *	0.048	3	0.403	0.070
4	0.077	0.739	4	−0.417	0.060	4	0.128	0.579	4	0.127	0.584
5	−0.023	0.920	5	−0.173	0.453	5	−0.232	0.311	5	−0.125	0.590
6	−0.189	0.411	6	0.104	0.653	6	−0.546 *	0.010	6	−0.312	0.168
7	−0.320	0.157	7	0.161	0.486	7	−0.720 **	0.000	7	−0.352	0.118

Note: CC: cross-correlation; * significance level 95%, ** significance level 99%.

**Table 2:** Lag correlation coefficient between public concern and O_3_ concentration for different ozone pollution peaks.

May 2016~June 2016 (Peak Time: 18 May)			May 2017~June 2017 (Peak Time: 28 May)			May 2018~June 2018 (Peak Time: 2 June)			May 2019~June 2019 (Peak Time: 24 May)		
Lag	CC	Std. Error	Lag	CC	Std. Error	Lag	CC	Std. Error	Lag	CC	Std. Error
−7	0.405	0.068	−7	0.207	0.369	−7	0.215	0.349	−7	0.076	0.743
−6	0.190	0.409	−6	−0.066	0.775	−6	0.055	0.814	−6	−0.195	0.397
−5	−0.077	0.739	−5	−0.304	0.180	−5	−0.204	0.375	−5	−0.077	0.741
−4	−0.320	0.157	−4	−0.360	0.109	−4	−0.123	0.596	−4	−0.027	0.907
−3	−0.306	0.177	−3	−0.423	0.056	−3	0.195	0.397	−3	−0.111	0.633
−2	−0.144	0.533	−2	−0.198	0.389	−2	0.337	0.135	−2	−0.143	0.536
−1	−0.056	0.810	−1	0.035	0.881	−1	0.324	0.152	−1	0.047	0.839
0	0.058	0.802	0	0.324	0.152	0	0.264	0.248	0	0.486 *	0.026
1	0.209	0.364	1	0.357	0.112	1	0.358	0.111	1	0.624 **	0.002
2	0.190	0.411	2	0.274	0.230	2	0.468 *	0.033	2	0.335	0.138
3	0.105	0.651	3	0.371	0.098	3	0.439 *	0.047	3	0.006	0.098
4	0.251	0.272	4	0.594 **	0.005	4	0.192	0.404	4	−0.161	0.485
5	0.479 *	0.028	5	0.533 *	0.013	5	−0.232	0.312	5	−0.281	0.217
6	0.615 **	0.003	6	0.369	0.100	6	−0.371	0.098	6	−0.357	0.112
7	0.484 *	0.026	7	0.252	0.271	7	−0.285	0.210	7	−0.366	0.103

Note: CC: cross-correlation; * significance level 95%, ** significance level 99%.

**Table 3:** Classification principles based on urban air quality and PI.

Quadrant	Air Quality Indicator	PI
Group I	C_PM2.5_ > 35 ug/m^3^	PI_haze_ > 0.239
	C_ozone_ > 160 ug/m^3^	PI_ozone_ > 0.268
	PP > 13.2%	PI > 0.259
Group II	C_PM2.5_ ≤ 35 ug/m^3^	PI_haze_ > 0.239
	C_ozone_ ≤ 160 ug/m^3^	PI_ozone_ > 0.268
	PP > 13.2%	PI > 0.259
Group III	C_PM2.5_ ≤ 35 ug/m^3^	PI_haze_ ≤ 0.239
	C_ozone_ ≤ 160 ug/m^3^	PI_ozone_ ≤ 0.268
	PP ≤ 13.2%	PI ≤ 0.259
Group IV	C_PM2.5_ > 35 ug/m^3^	PI_haze_ ≤ 0.239
	C_ozone_ > 160 ug/m^3^	PI_ozone_ ≤ 0.268
	PP > 13.2%	PI ≤ 0.259

Note: PP represents the proportion of polluted days; 0.239 is the median value of all the values of PIhaze; 0.268, 0.259, and 13.2% are the median values of all PIozone, PI, and PP values, respectively.

## Data Availability

The data are not publicly available, and any inquiries may be addressed by the first author.
